# Human liver tissue transcriptomics revealed immunometabolic disturbances and related biomarkers in hepatitis B virus-related acute-on-chronic liver failure

**DOI:** 10.3389/fmicb.2022.1080484

**Published:** 2022-12-01

**Authors:** Luo Yang, Limin Zhen, Zhihui Li, Shu Zhu, Wenxiong Xu, Qiumin Luo, Liang Peng, Chan Xie

**Affiliations:** ^1^Department of Infectious Diseases, Third Affiliated Hospital of Sun Yat-sen University, Guangzhou, China; ^2^Guangdong Provincial Key Laboratory of Liver Disease Research, Guangzhou, Guangdong, China; ^3^Key Laboratory of Tropical Diseases Control, Ministry of Education, Guangzhou, China

**Keywords:** human liver, transcriptomics, immunometabolic, disturbances, HBV-ACLF

## Abstract

Acute-on-chronic liver failure (ACLF) is a major cause of liver-related death worldwide, but its key pathological features remain incompletely defined. This study aimed to reveal the molecular basis of hepatitis B virus-related ACLF (HBV-ACLF) by transcriptome sequencing of human liver tissue. A total of 18 human liver tissues from patients with different stages of HBV-related disease were collected for RNA sequencing, and liver tissues from patients and mouse models with ACLF were used for subsequent validation. Specifically, 6,853 differentially expressed genes (DEGs) and 5,038 differentially expressed transcripts were identified in patients with ACLF compared to patients with chronic hepatitis B (CHB) and normal controls (NCs). Investigation of functional by KEGG pathway enrichment analysis revealed prominent immune and metabolic dysregulation at the ACLF stage. We found that the key genes FGF19, ADCY8 and KRT17, which are related to immunometabolic disturbances, were significantly upregulated in the progression of ACLF. The three key genes were validated in human and mouse samples, indicating their prognostic and therapeutic potential in ACLF. In summary, our work reveals that immunometabolic disorder is involved in HBV-ACLF pathogenesis and indicates that FGF19, ADCY8 and KRT17 may be sensitive biomarkers for HBV-related ACLF.

## Introduction

Acute-on-chronic liver failure (ACLF) is a complex clinical syndrome characterized by rapid deterioration of chronic liver disease accompanied by organ failure and a high short-term mortality rate of 50–90% (Arroyo et al., 2020). Hepatitis B virus (HBV), is associated with acute and chronic hepatitis and hepatocellular carcinoma. ACLF occurs in approximately 30% of patients with HBV-related liver disease and has a poor short-term prognosis (Zheng et al., 2011; Shi et al., 2015). Understanding the molecular mechanism of HBV-ACLF is crucial for the diagnosis and development of effective treatment strategies. Liver failure is related mainly to systemic inflammatory responses (Moreau et al., 2013; Clària et al., 2016; Trebicka et al., 2021). Jorn M S showed that cell death and inflammation driven by the IL-1/IL-1R1 pathway can lead to liver failure (Gehrke et al., 2018). In addition, IL-23R expressed in Th17 cells of ACLF patients can induce inflammation and is closely related to the severity of liver failure (Khanam et al., 2019). However, both immunity and metabolism are essential for maintaining liver homeostasis. Immunometabolic disorders can lead to pathological changes in the liver, characterized by progressive to hepatitis, fibrosis, cirrhosis, liver failure, and liver cancer. Impairment of liver macrophages in patients with cirrhosis has been reported to be related to the severity of liver disease (Irvine et al., 2019). Immunometabolic disorders also play an important role in the proliferation, differentiation, and function of cancer cells. However, the role of immunometabolic abnormalities in ACLF has rarely been reported.

A transcriptome is a collection of all RNA transcribed from a particular tissue or cell in a certain developmental stage or functional state. New strategies for disease diagnosis and treatment can be developed by decoding genome structure and function, rebuilding gene interaction networks and identifying new biomarkers (Jiang et al., 2015). Previous animal studies based on transcriptional profiling revealed that the progression of liver failure was related to the immune response (Zhang et al., 2018; Hassan et al., 2022). Furthermore, transcriptomic studies of human peripheral blood mononuclear cells (PBMCs) showed that immunometabolic disorders constitute the core axis of ACLF development (Li et al., 2022). Since human liver tissue is difficult to obtain, the above study only suggested the role of immunometabolism in liver failure via limited pathways, and direct clinical and transcriptomic evidence based on human liver tissue remains lacking. In this study, we directly collected liver tissues of HBV-ACLF patients at different disease stages for transcriptome sequencing and further elucidated the role of immunometabolic disorders and the detailed molecular mechanisms in the progression of HBV-ACLF. Based on Kyoto Encyclopedia of Genes and Genomes (KEGG) database and gene set enrichment analysis (GSEA), we identified 3 key genes, fibroblast growth factor 19 (FGF19) (and its mouse ortholog Fgf15), adenylate cyclase 8 (ADCY8) and keratin 17 (KER17), thus providing a more direct clinical basis for the identification of biomarkers for HBV-ACLF.

## Materials and methods

### Study design and definitions

**FIGURE 1:**
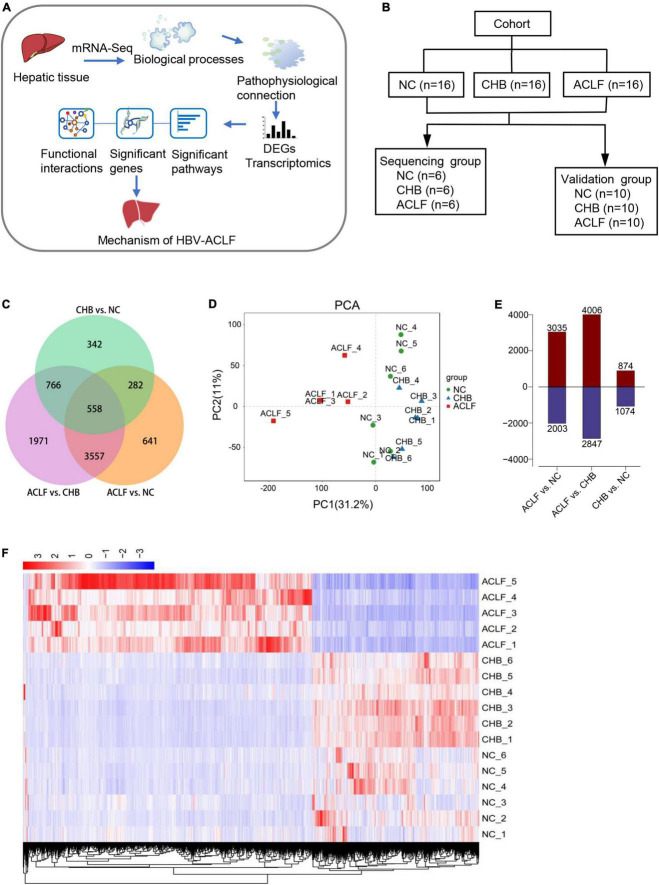
Transcriptomic features in patients with HBV-ACLF. **(A)** Flow diagram of the strategy used for analysis of functional synergy. **(B)** Study design and patient group allocation. **(C)** Venn diagram of the DEGs analyzed in pairwise comparisons among subjects in the acute-on-chronic liver failure (ACLF), chronic hepatitis B (CHB) and normal control (NC) groups. Numbers of mRNA sequences (*n* = 5/6/6, ACLF/CHB/NC groups). **(D)** PCA of subjects in the ACLF, CHB, and NC groups. **(E)** Numbers of significant DEGs in the pairwise comparisons. **(F)** Distribution of the 4115 DEGs in HBV-ACLF, CHB and NC subjects. HBV-ACLF, HBV-related acute-on-chronic liver failure; CHB, chronic hepatitis B; NC, normal control; DEGs, differentially expressed genes.

To elucidate the role and detailed molecular mechanism of immunometabolic disorders in HBV-ACLF disease progression, we collected fresh liver tissues from patients with different stages of HBV-related disease for mRNA sequencing (mRNA-seq) and subsequent validation. Functional synergy was then analyzed to identify variations in genes and biological processes associated with the clinical pathophysiology of HBV-ACLF (Figure 1A). Samples of fresh liver tissue from 16 normal controls (NCs), 16 patients with CHB and 16 patients with HBV-ACLF were collected and randomly divided into a sequencing cohort and validation cohort. The sequencing cohort consisted of 6 patients from each group and was used for RNA sequencing. The validation cohort consisted of 10 patients in each group and was used for subsequent qPCR and immunohistochemical (IHC) validation (Figure 1B). Patients with concomitant diseases were excluded.

The diagnosis of chronic HBV infection was based on seropositivity for hepatitis B surface antigen (HBsAg) and/or HBV-DNA for more than 6 months in accordance with the Guidelines for the Prevention and Treatment of Chronic Hepatitis B (2019 version). ACLF constitutes an acute deterioration based on known or unknown chronic liver disease or cirrhosis and is defined as severe liver injury resulting in coagulation abnormalities, usually with an international normalized ratio (INR) ≥ 1.5 and any degree of mental change (Sarin et al., 2019). The NCs were healthy volunteers (aged 18–60 years) who were medically qualified donors for liver transplantation.

### Human liver tissue dissociation

Sixteen normal liver tissues were obtained from donor livers intended for transplantation. Sixteen CHB liver tissues were obtained through percutaneous or transjugular liver biopsy. Sixteen HBV-ACLF liver tissues were obtained from patients during surgery before any therapeutic intervention. Liver samples were removed, snap-frozen in liquid nitrogen, and then stored for mRNA-seq. Written informed consent was received from the study subjects or the legal representatives of the patients prior to inclusion in the study, and the acquisition of these samples was approved by the Clinical Research Ethics Committee of the Third Affiliated Hospital of Sun Yat-sen University.

### Establishment of mouse acute-on-chronic liver failure models

We established mouse models of acute-on-chronic liver injury by combining chronic injury and acute hepatic insult to mimic ACLF conditions. Six-week-old male C57BL/6J mice were used for all experiments. All mice were randomly allocated to the groups. The CCl_4_-induced liver cirrhosis model (LC) was established via intraperitoneal injection of 10% CCl_4_ (Macklin C805332, Shanghai, China) dissolved in an olive oil solution (Macklin O815211, Shanghai, China) (5 ml/kg body weight) twice weekly for 8 weeks. Mice in the ALCF group were injected with 50% CCl_4_ (8 ml/kg body weight) in the last injection based on the stage of cirrhosis and were killed 6 h later. Mice in the control group were intraperitoneally injected with only olive oil (5 ml/kg body weight) twice weekly for 8 weeks. Six animals per group were used for the above studies. The levels of ALT, AST, TBIL, and ALB were measured on a Hitachi 7600 automatic biochemical analyzer.

### mRNA-sequencing, quantitative real-time-polymerase chain reaction and histological staining

Total RNA was isolated from frozen liver samples using TRIzol reagent (15596018, Thermo Fisher Scientific, Inc., Waltham, MA, USA). mRNA-seq was performed using a TruSeq RNA Sample Preparation Kit V2 (Illumina, San Diego, CA, USA). Three potential biomarkers were validated by qRT-PCR and IHC staining in the validation cohort and mouse ACLF model. Hematoxylin-eosin (H&E), Masson’s trichrome (M&T) and Sirius red (S&R) staining were performed according to the kit instructions. Information about the materials, primers, and antibodies used for qRT-PCR and IHC staining is provided in Supplementary Tables 1, 2 in the online Supplementary material.

### Enzyme-linked immunosorbent assay

Human serum FGF19 levels were determined using enzyme-linked immunosorbent assay (ELISA) kits (Multiscience, Hangzhou, China); Human serum ADCY8 levels were determined using enzyme-linked immunosorbent assay (ELISA) kits (Bioswamp, Wuhan, China); Human serum KRT17 levels were determined using enzyme-linked immunosorbent assay (ELISA) kits (Cloud-Clone Corp., Wuhan, China).

### Processing of liver transcriptome datasets

All samples were tested for total RNA quality. Then, mRNA was purified and fragmented, first-strand and second-strand cDNA were synthesized, and the double-stranded cDNA was subjected to end repair, adenylation of 3’ ends, and adapter ligation. After size selection, the DNA libraries were amplified, quality controlled, and subjected to sequencing. Raw data were collated, filtered, and subjected to quality assessment. Quantitative analysis of mRNA expression and differential mRNA expression analysis were then performed. Significant DEGs were identified and clustered into biological processes through network-based analysis of functional synergy. KEGG pathway enrichment analysis was performed with GSEA software^1^ to analyze biological significance.

### Statistical analysis

GraphPad Prism 8 (San Diego, CA, USA) was used for statistical analysis. All values are expressed as the means ± SEMs. We determined statistical significance by one-way ANOVA for multiple group analyses. A *p* value < 0.05 was considered statistically significant. *P < 0.05, **P < 0.01, ***P < 0.001.

## Results

### Clinical characteristics of the enrolled subjects

A total of 16 patients with HBV-ACLF, 16 patients with chronic hepatitis B and 16 NCs were enrolled in this study. A total of 18 were assigned to the sequencing cohort (6 subjects each from the HBV-ACLF, CHB and NC groups), and 30 (10 subjects per group) were assigned to the validation cohort. Patients with concomitant diseases were excluded. All HBV-ACLF patients were diagnosed with cirrhosis, and all CHB patients were outpatients who were in the inactive disease phase. Normal livers were obtained from healthy liver donors undergoing hepatic resection, and the corresponding samples were considered the NC group in this study. Table 1 shows the demographic and clinical characteristics of the HBV-ACLF patients, CHB patients and NC group at admission. The levels of serum aspartate aminotransferase (AST), alanine aminotransferase (ALT), alkaline phosphatase (ALP), total bilirubin (TBILI), gamma-glutamyl transferase (GGT), creatinine, PT (prothrombin time) and INR (international normalized ratio) were significantly higher and albumin (ALB), hemoglobin, and platelet count were significantly lower in the ACLF group than in the CHB/NC groups. These findings indicate ACLF with severe liver failure and coagulation failure. Among the 18 subjects in the sequencing group, one sample from a patient with HBV-ACLF did not reach the quality check criterion and was excluded from this study.

**TABLE 1:** Baseline participant characteristics.

Variable	HBV-ACLF (*n* = 16)	CHB (*n* = 16)	NC (*n* = 16)
Age (years)	43.5 (39.25, 48.25)	36.0 (31.75, 43.5)	50.0 (42.75, 53.25)
Sex (M/F)	(14/2)	(12/4)	(5/11)
Male gender	14 (87.5%)	12 (75.0%)	5 (31.3%)
Female gender	2 (12.5%)	4 (25.0%)	11 (68.7%)
HBV-DNAlog_10_ (IU/ml)	4.8 (2.2, 5.7)	4.2 (3.8, 5.3)	NA
AST (U/L)	123.0 (79.5, 225.5)	24.5 (22.0, 33.5)***	20.5 (18.0, 23.2)***
ALT (U/L)	73.0 (51.8, 142.0)	37.0 (26.0, 62.5)*	16.0 (15.0, 18.0)**
Albumin (g/L)	38.5 (36.2, 41.2)	47.3 (46.6, 49.6)***	43.7 (42.1, 46.4)***
ALP (U/L)	114.5 (97.0, 138.5)	57.0 (52.5, 67)***	48.5 (45.0, 57.8)***
Total bilirubin (μmol/L)	32.3 (26.7, 33.6)	10.9 (7.3, 15.5)***	8.9 (5.6, 16.5)***
GGT (U/L)	57.0 (36.0, 100.3)	20.5 (17.0, 27.0)***	20.5 (16.8, 27.8)***
Creatinine (μmol/L)	81.5 (73.0, 94.0)	71 (59.5, 81.5)*	54 (51.8, 57.8)***
WBC count (10^9^/L)	5.9 (5.1, 9.9)	6.5 (5.2, 7.4)	4.8 (4.5, 5.3)*
Hemoglobin (g/L)	97.5 (87.8, 117.5)	149.5 (140.8, 159.0)***	128 (119.8, 133.3)***
Platelet count (10^9^/L)	85.5 (54.8, 178.5)	219.5 (201.5, 265.3)***	222.5 (198.8, 251.8)***
PT (s)	33.9 (31.0, 40.9)	13.1 (12.6, 13.4)***	12.8 (12.6, 13.0)***
INR	3.3 (3.0, 4.2)	1.0 (0.9, 1.0)***	1.0 (0.9, 1.0)***
AFP (μg/L)	5.3 (4.1, 13.8)	2.9 (2.1, 4.4)	2.7 (2.1, 4.3)
COSSH-ACLFs	8.5 (7.7, 10.2)	NA	NA
CLIF-C ACLFs	31.1 (27.4, 37.6)	NA	NA
MELD	33.3 (29.3, 36.1)	NA	NA

All summary data are presented as the medians (p25, p75) or sums with proportions.

*p < 0.05, **p < 0.01 and ***p < 0.001 for comparisons between the CHB/NC and ACLF groups.

AST, aspartate aminotransferase; ALT, alanine aminotransferase; ALP, alkaline phosphatase; GGT, gamma-glutamyl transferase; WBC, wide blood cell; PT, prothrombin time; INR, international normalized ratio; AFP, Alpha fetoprotein; ACLF, acute-on-chronic liver failure; CHB, chronic hepatitis B; NC, normal controls. COSSH-ACLFs, Chinese Group on the Study of Severe Hepatitis B-ACLF score; CLIF-C ACLFs, Chronic Liver Failure Consortium ACLF score; MELD, model for end-stage liver disease; NA, not applicable.

### Transcriptomic characteristics of liver tissues

The DEGs in the transcriptome of patients with HBV-ACLF compared with the transcriptomes of patients with CHB and NCs were analyzed using R (version 3.6.3) and R packages. This analysis indicated that 6,853 and 5,038 genes were significantly differentially expressed in patients with HBV-ACLF compared with patients with CHB and NCs, respectively (Figure 1C). Principal component analysis (PCA) of gene expression (performed by Omicshare^2^) in liver tissues showed significant differences between the ACLF, CHB, and NC groups (Figure 1D), indicating considerable changes in the transcriptome profile during ACLF progression. As indicated in Figure 1E, the numbers of significant DEGs were increased in the ACLF group compared with the CHB and NC groups (4,006 upregulated/2,847 downregulated and 3,035 upregulated/2,003 downregulated, respectively). However, only 1,948 genes were markedly differentially expressed between the CHB and NC groups (874 upregulated/1074 downregulated). To explore the transcriptome expression pattern in liver tissue, unsupervised stratified cluster analysis was performed based on the DEGs between multiple groups. Pairwise differential expression analyses were performed using a false discovery rate (FDR) threshold of less than 0.05. DEGs from the two comparison groups (ACLF/CHB and ACLF/NC) were intersected, resulting in a group of 4115 DEGs in the HBV-ACLF group. Cluster analysis based on the normalized expression of these 4115 genes showed that all HBV-ACLF patients were clustered together, suggesting that these DEGs could be used to distinguish patients with HBV-ACLF from patients with CHB and NCs (Figure 1F). Analysis of the NC, CHB and ACLF groups showed that ACLF was a stage dissimilar to CHB and normal health (i.e., the NC group); therefore, these two groups could be used as controls for subsequent analyses.

### Transcriptome analysis revealed the role of immunometabolism in patients with acute-on-chronic liver failure

**FIGURE 2:**
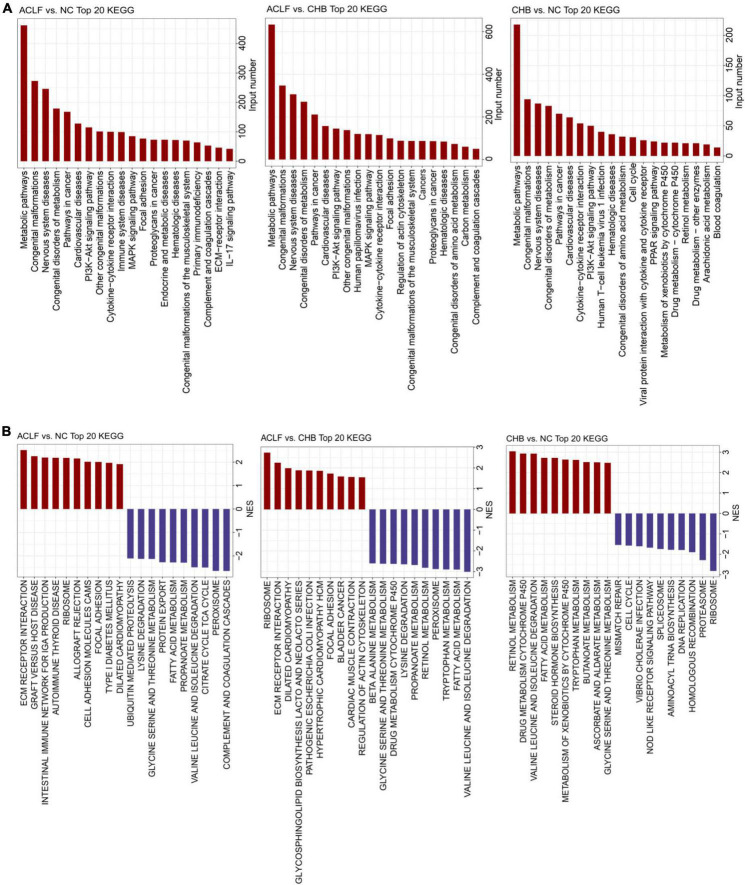
Functional alterations during acute-on-chronic liver failure (ACLF) development. **(A)** Bar chart of KEGG pathway enrichment analysis of the DEGs showing the top 20 enriched KEGG pathways. **(B)** Twenty significantly upregulated and downregulated biological processes were identified using GSEA in pairwise comparisons among the three groups. KEGG, Kyoto Encyclopedia of Genes and Genomes; GSEA, gene set enrichment analysis.

To identify the key pathophysiological variations that occur during ACLF progression, KEGG pathway enrichment analysis and GSEA were performed based on the significantly correlated genes. The top 20 significantly differentially regulated pathways were identified by the minimum adjusted *p* values in pairwise functional analysis. Metabolism-related pathways (pathways in cancer, metabolic pathways, MAPK signaling pathway, congenital disorders of metabolism, and PI3K-Akt signaling pathway) and immunity-related pathways (immune system diseases, primary immunodeficiency, IL-17 signaling pathway, complement and coagulation cascades, and cytokine-cytokine receptor interaction) were significantly enriched across the pairwise comparisons among the three groups (Figure 2A). We further found that metabolism-related pathways (valine, leucine and isoleucine degradation; fatty acid metabolism; peroxisome; propanoate metabolism; lysine degradation; and glycine, serine and threonine metabolism) were enriched with the downregulated genes, whereas immune-related pathways (ECM receptor interaction, graft-versus-host disease, cell adhesion molecules (CAMs), autoimmune thyroid disease and intestinal immune network for production) were enriched with the upregulated genes in the ACLF group compared with the CHB and NC groups (Figure 2B). The results suggested that immune and metabolic alterations may play a key role in the pathophysiological process of ACLF progression.

### Identification of key genes in hepatitis B virus-related acute-on-chronic liver failure patients

**FIGURE 3:**
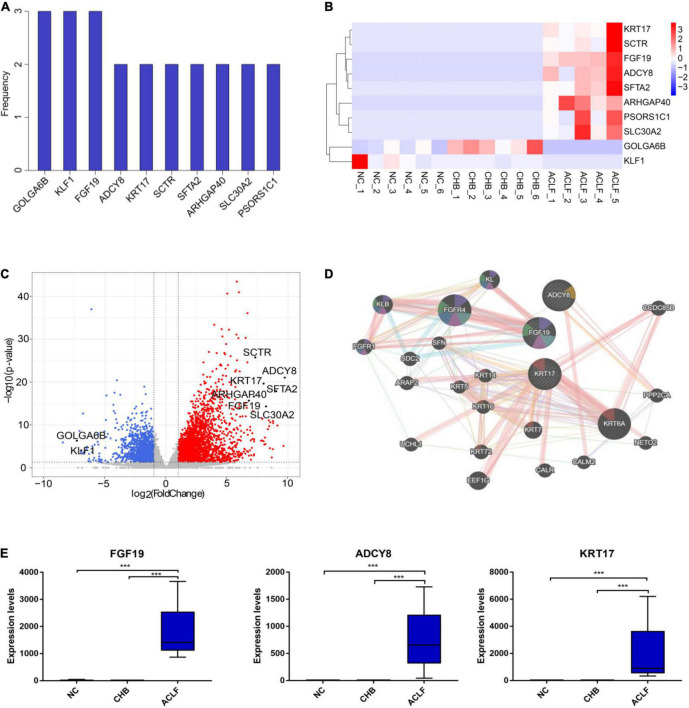
Genes associated with immunometabolism in HBV-ACLF development. **(A)** The frequencies of the top 10 genes among the top 50 DEGs from the three comparisons (ranked by adjusted *p* value). **(B)** Heatmap of 10 DEGs in the three groups. **(C)** Volcano plot showing the top 10 DEGs in the comparison of the ACLF and NC groups. Red and blue indicate significant DEGs (| log2-fold change| > 1.5; FDR < 0.05). **(D)** GeneMANIA (http://www.genemania.org) showing the genes interacting with the top three genes (fibroblast growth factor 19, FGF19; adenylate cyclase 8, ADCY8; keratin 17, KRT17). **(E)** The expression of the three genes in the sequencing cohort. ***Adjusted *P* value < 0.001. HBV, hepatitis B virus; ACLF, acute-on-chronic liver failure; CHB, chronic hepatitis B; NC, normal control; DEGs, differentially expressed genes; FGF19, fibroblast growth factor 19; ADCY8, adenylate cyclase 8; KRT17, keratin 17.

We next conducted three pairwise comparisons between disease stages (among the NC, CHB and ACLF groups) to identify key molecules that were differentially expressed and correlated with the pathophysiology of ACLF. The frequencies of occurrence of the top 50 DEGs in the three comparisons were calculated (Figure 3A). A heatmap was generated to show the expression of the top 10 DEGs in three groups of seventeen samples selected by frequency and adjusted *p* value (Figure 3B). In addition, the volcano plot showed that both GOLGA6B and KLF1 were downregulated in the ACLF group compared with the NC and CHB groups, whereas FGF19, ADCY8, KRT17, SCTR, SFTA2, ARHGAP40, SLC30A2 and PSORS1C1 were upregulated in the ACLF group compared with the CHB and NC groups (Figure 3C). From the three comparisons among the groups, we found that the expression of FGF19 was markedly increased in the ACLF group. In addition, ADCY8 and KRT17 exhibited the two lowest adjusted *p* values in the comparison between the ACLF and NC groups. GeneMANIA^3^ was used to visualize the interconnections among the three genes (Warde-Farley et al., 2010) (Figure 3D). The results of qRT-PCR in the sequencing cohort showed increased expression of FGF19, ADCY8 and KRT17 during the progression of ACLF (Figure 3E). By DEG analysis of the transcriptome data, we identified the key genes FGF19, ADCY8 and KRT17, which might be further developed as potential diagnostic and prognostic biomarkers for ACLF.

### FGF19, ADCY8 and KRT17 are associated with immunometabolic disorders

**FIGURE 4:**
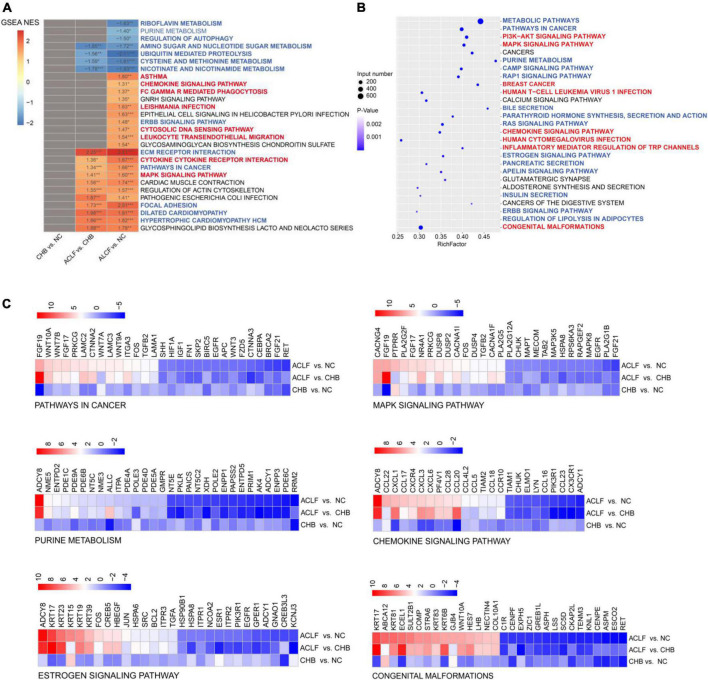
Immunometabolic pathways (containing the three target genes) were significantly altered in the development of ACLF. **(A)** Enrichment analysis was performed using GSEA, with the red and blue numbers indicating the normalized enrichment score for each gene set. The color intensity is proportional to the NES calculated using GSEA. Immunometabolic pathways were significantly altered during the development of ACLF, based on the enriched KEGG pathways. Blue and red highlighting indicate metabolic and immune pathways, respectively. **(B)** Bubble plot of the three target genes significantly associated with the 27 immunometabolic KEGG pathways, with blue and red highlighting indicating metabolic and immune pathways, respectively. **(C)** Heatmap of three specific biomarkers from the selected immunometabolic pathways. The top 30 DEGs in each pathway (adjusted *p* value < 0.05) are shown. Genes ranked less than 30 are shown completely. Genes with higher (red) or lower (blue) expression are shown proportionally. ACLF, acute-on-chronic liver failure; HBV, hepatitis B virus; CHB, chronic hepatitis B; NC, normal control; GSEA, gene set enrichment analysis; NES, normalized enrichment score; KEGG, Kyoto Encyclopedia of Genes and Genomes.

The ACLF immunometabolic pathways from the KEGG database were used for functional annotation based on the GSEA results. The top twenty-eight pathways (ranked by *p* value, *p* < 0.05) were enriched across ACLF development (Figure 4A), and most were immunometabolism-related. Twenty-one of these pathways were upregulated and seven were downregulated in the ACLF group. Immunometabolic pathways were significantly changed (blue and red highlighting indicate downregulated metabolism-related pathways and upregulated immune-related pathways, respectively) (*p* < 0.0001), including ubiquitin-mediated proteolysis, cysteine and methionine metabolism, nicotinate and nicotinamide metabolism, leukocyte transendothelial migration, ECM receptor interaction, cytokine–cytokine receptor interaction, pathways in cancer, MAPK signaling pathway, focal adhesion, dilated cardiomyopathy, and hypertrophic cardiomyopathy (HCM). These pathways play roles in proteolysis, amino acid metabolism, cell growth, cell differentiation, stress, inflammatory responses and carcinogenesis. We then performed KEGG pathway enrichment analysis of the pathways containing FGF19, ADCY8 and KRT17. A total of 27 common pathways were identified by pairwise comparisons among the three groups (*p* < 0.0001); most of these pathways were related to immunometabolism (Figure 4B). Moreover, FGF19, ADCY8 and KRT17 were involved in biological processes such as regulation of actin cytoskeleton, pathways in cancer, MAPK signaling pathway, dilated cardiomyopathy, chemokine signaling pathway, purine metabolism gnrh signaling pathway, estrogen signaling pathway and congenital malformations (Figure 4C), all of which are related to immunological disturbances and metabolic disorders. Together, these findings show that FGF19, ADCY8 and KRT17 play important pathophysiological roles in the progression of ACLF and are associated with immunometabolic disorders.

### Validation of FGF19, ADCY8 and KRT17 in amplified human samples

**FIGURE 5:**
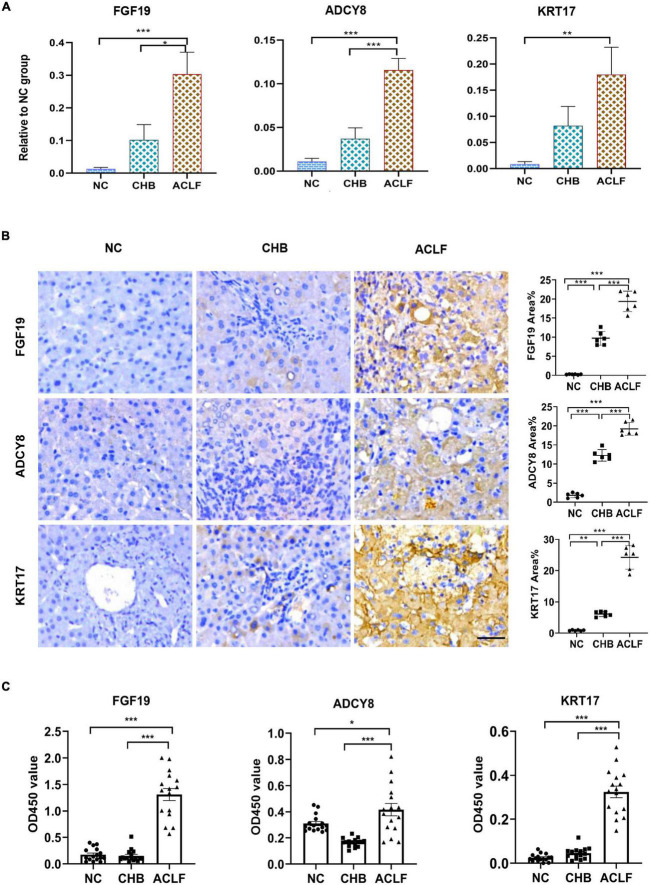
External validation by qRT-PCR and IHC staining in amplified human samples. **(A)** qRT-PCR validation of three genes (*n* = 10/10/10, NC/CHB/ACLF groups). **(B)** IHC and quantification of three key proteins involved in immunometabolic disorder in patients in the NC, CHB and HBV-ACLF validation cohort (bar = 50 μ*m*). **(C)** Content of three markers in human serum (*n* = 16/16/16, NC/CHB/ACLF, respectively). The values are presented as the means ± SEMs. *Adjusted *p* < 0.05, **Adjusted *p* < 0.01, ***Adjusted *p* < 0.001. HBV, hepatitis B virus; ACLF, acute-on-chronic liver failure; CHB, chronic hepatitis B; NC, normal control; FGF19, fibroblast growth factor 19; ADCY8, adenylate cyclase 8; KRT17, keratin 17; IHC, immunohistochemical.

The three key molecules associated with immunometabolic disorders were validated in the external validation cohort. The mRNA levels of the three genes were increased in the order of NC to CHB to ACLF (Figure 5A). IHC staining showed that the expression of the three corresponding proteins was also increased during disease progression from NC to CHB to ACLF (Figure 5B), consistent with the sequencing results. Serum FGF19, ADCY8, KRT17 levels were determined using enzyme-linked immunosorbent assay (ELISA) kits. The results showed that the contents of the three markers in the ACLF group were significantly higher than those in the NC and CHB groups (Figure 5C). These findings highlighted the relevance of these molecules to human ACLF and suggest their diagnostic and prognostic potential in ACLF.

### Validation of FGF15, ADCY8 and KRT17 in acute-on-chronic liver failure mouse models

**FIGURE 6:**
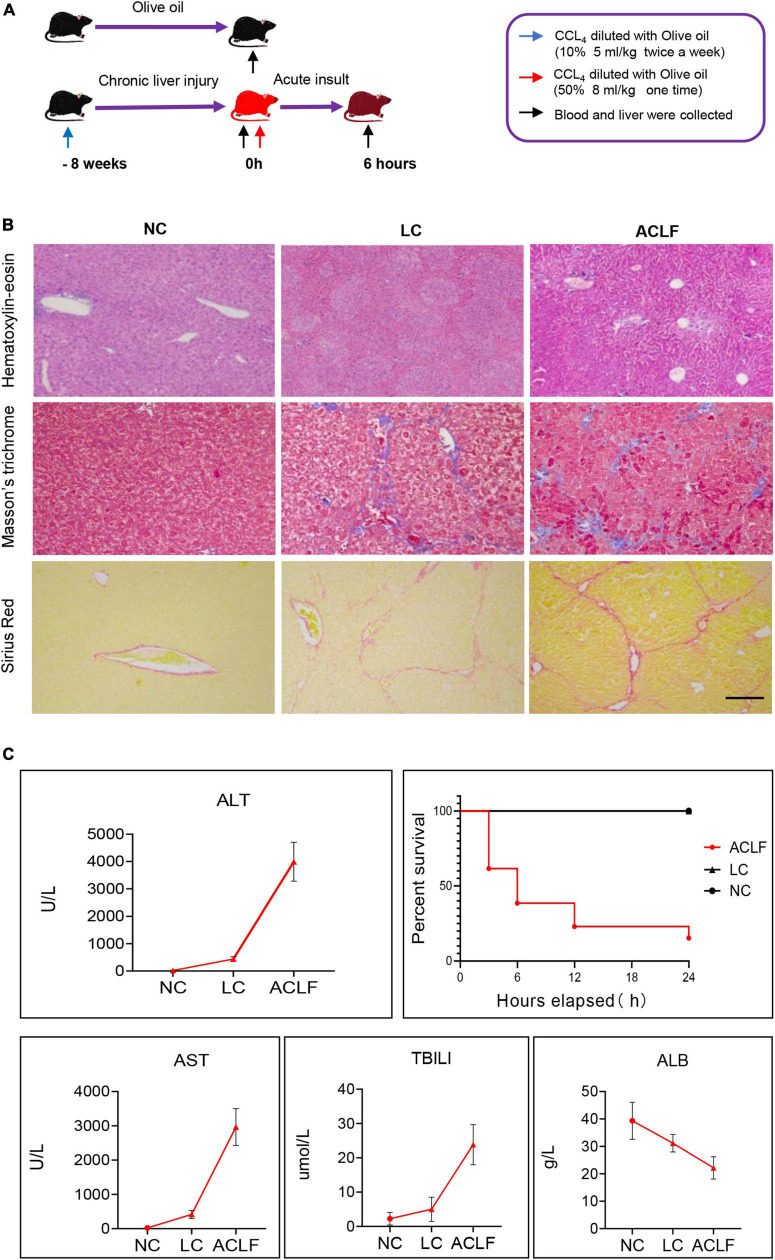
Establishment of the acute-on-chronic liver failure mouse model. **(A)** Schematic diagram of the mouse ACLF model. **(B)** Pathological staining (H&E, M&T and S&R) of liver tissues at different disease stages (bar = 100 μm). **(C)** Four typical biochemical markers of liver function were evaluated in the three groups (*n* = 6/6/6, NC/CHB/ACLF groups), and mortality was observed in the ACLF group. ACLF, acute-on-chronic liver failure; LC, liver cirrhosis; NC, normal control; H&E, hematoxylin-eosin; M&T, Masson’s trichrome; S&R, Sirius red; ALT, alanine aminotransferase; AST, aspartate aminotransferase; TBILI, total bilirubin; ALB, albumin.

To further confirm the roles of FGF15, ADCY8 and KRT17 in ACLF, we established mouse models of acute-on-chronic liver injury (Figure 6A). H&E, M&T and S&R staining revealed the typical histology of cirrhosis-based liver failure, including extensive hepatocyte inflammation, necrosis, collagen accumulation, fibrous hyperplasia and abnormal hepatic lobule structure (Figure 6B). Liver function analysis showed significantly elevated serum ALT, AST, and TBILI and decreased levels of ALB in ACLF mice (Figure 6C). The 6-, 12-, and 24-h mortality rates in the liver failure group were 61.54, 77, and 84.6%, respectively (Figure 6C).

**FIGURE 7:**
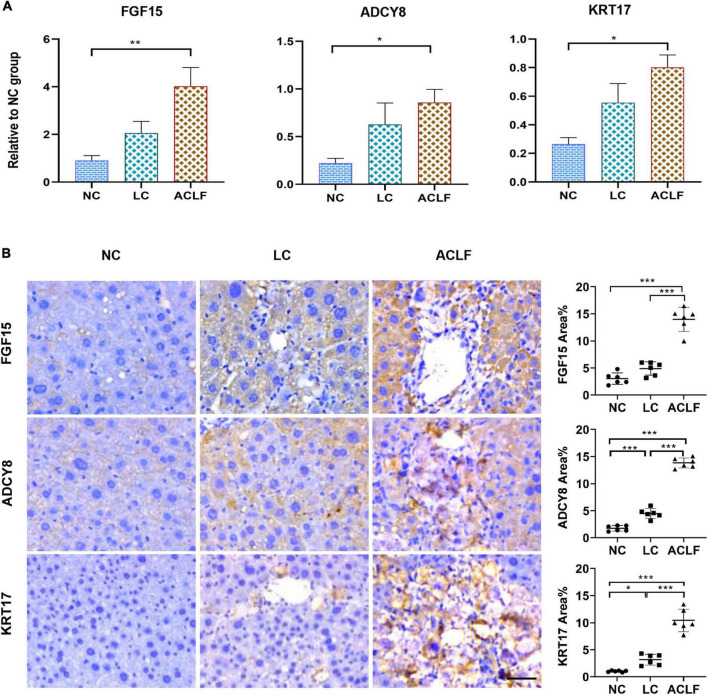
Validation of the three biomarkers related to immunometabolic disorder in ACLF mouse model. **(A)** qRT-PCR validation of three key genes (*n* = 6/6/6, ACLF/CHB/NC groups). The values are presented as the means ± SEMs. *Adjusted *p* < 0.05, **Adjusted *p* < 0.01, ***Adjusted *p* < 0.001. **(B)** Representative images of IHC staining and quantification of the three key proteins involved in ACLF development in the ACLF, LC and NC groups in the mouse model (bar = 50 μm). The values are presented as the means ± SEMs. *Adjusted *p* < 0.05, **Adjusted *p* < 0.01, ***Adjusted *p* < 0.001. HBV, hepatitis B virus; ACLF, acute-on-chronic liver failure; CHB, chronic hepatitis B; NC, normal control; FGF15, fibroblast growth factor 15; ADCY8, adenylate cyclase 8; KRT17, keratin 17.

The three potential biomarkers associated with immunometabolic disorders related to ACLF development were also validated in three groups of mouse liver tissues by qRT-PCR and IHC staining, and the results showed gradually increasing expression of FGF15, ADCY8 and KRT17 from NC and LC to ACLF mice (Figure 7A). IHC staining showed a gradual increase in the expression of the FGF15, ADCY8 and KRT17 proteins from NC and LC to ACLF mice (Figure 7B). These patterns in the mouse models were similar to those in patients with liver failure, further confirming the sensitivity of the three molecules for characterizing HBV-ACLF progression.

## Discussion

To intuitively understand the pathological changes in the progression of ACLF, we collected human liver tissue at different stages of disease for transcriptomic sequencing to reveal the molecular mechanism.

In this study, transcriptome analysis suggested that immunometabolic disorders are the core mechanism driving ACLF development and prognosis. KEGG pathway enrichment analysis of the DEGs in the ACLF, CHB and NC groups revealed that immunometabolic pathways were significantly enriched during the progression of ACLF. Specifically, metabolism-related pathways (including amino acid metabolism, fatty acid metabolism, and peroxisome metabolism) were downregulated in the ACLF group compared with the CHB and NC groups. Studies have confirmed that liver failure is associated with reduced circulating lipid levels and hepatic lipid accumulation (Bollard et al., 2010). Metabolic abnormalities are also associated with increased mortality and severity in patients with liver diseases (Kyriakides et al., 2014). We also observed that immune-related pathways, including immune system diseases, intestinal immune network for production, primary immunodeficiency, IL-17 signaling pathway, complement and coagulation cascades and cytokine-cytokine receptor interaction, were upregulated in the ACLF group. HBV infection triggers inflammation as well as immune responses. Dysfunction of the innate and adaptive immune systems also induces cirrhosis, an important intermediate process in the development of ACLF (Riva and Mehta, 2019). In addition, immunological abnormalities in the intestinal flora have been associated with the development of ACLF, consistent with our results regarding enrichment of a pathway associated with intestinal immune abnormalities in the ACLF group. These results indicate that immunometabolic processes drive the development of HBV-ACLF.

We next sought to further identify the immunometabolic pathway-related biomarkers involved in the progression of HBV-ACLF. Based on transcriptome data and clinical presentation-oriented analysis of functional synergy, we identified three potential biomarkers (FGF19, ADCY8 and KRT17) that primarily reflected the HBV-related immunometabolic disorders during ACLF. FGF19 (and its mouse ortholog Fgf15) is a member of a fibroblast growth factor subfamily that regulates various metabolic pathways. FGF19 is involved in the homeostatic control of bile acid, carbohydrate, and lipid metabolism in multiple target organs, including the liver and adipose tissue. Humans FGF19 expression is regulated by bile acids, and FGF19 can also repress bile acid synthesis and prevent subsequent liver damage. Dysregulation of FGF19 contributes to many metabolism-associated diseases, such as fatty liver disease and type 2 diabetes. FGF19 could also be a new therapeutic target for metabolic diseases (Bozadjieva et al., 2018). More importantly, consistent with our findings, FGF19 has been reported to be associated with the development of liver diseases, including HBV-related cirrhosis and hepatocellular carcinoma (Gorgoulis et al., 2000; Ahn et al., 2014; Raja et al., 2019), possibly via FGF19-FGFR4 pathway-related immune dysregulation (Repetto et al., 2021). ADCY8 is a membrane-binding enzyme that catalyzes the formation of cyclic AMP from ATP. Studies have shown that ADCY8 induces innate immune-associated antiviral host defense in response to macrophage SCN5A (Jones et al., 2014), and ADCY8 also affects glucagon-like peptide 1 signaling and glucose levels in metabolic diseases (Roger et al., 2010).

Keratin 17 (KRT17), a type I intermediate filament, regulates many biological processes and signaling for protein synthesis and cell growth. KRT17 blocks T-cell infiltration to induce an inflammatory microenvironment and immune disorder (Markey et al., 1992; Depianto et al., 2010). Upregulated KRT17 promotes hepatic stellate cell (HSC) activation, which is a potential therapeutic target in liver fibrosis (Chen et al., 2022). In the current study, consistent with the reports regarding the roles of these three molecules in immunometabolic pathways and liver diseases, our data based on liver tissues from human patients with ACLF validated that FGF19, ADCY8 and KRT17 are upregulated in ACLF and associated with immunometabolic disorder, suggesting that FGF19, ADCY8 and KRT17 could be sensitive biomarkers for the diagnosis of HBV-ALCF. In addition, we drew on the ACLF animal model of published journalsto construct HBV-free LC and ACLF mouse models with CCl4 and once again verified the role of three biomarkers in the process of liver failure (Li et al., 2022). Unfortunately, since there is currently no suitable HBV-ACLF animal model, we will pay attention to improving this model in future studies.

In summary, transcriptome sequencing of human liver tissues revealed the relationships between FGF19, ADCY8 and KRT17 and immunometabolic processes, defining unique characteristics in the development of ACLF. We provided new insight into the design of FGF19-, ADCY8- and KRT17-targeted inhibitors for ACLF treatment. In the future, the in-depth mechanism by which these three genes individually or cooperatively influence immunometabolic processes to promote ACLF will be explored through more exhaustive molecular studies and clinical validation based on a larger cohort of HBV-ACLF patients and detailed data.

## Data availability statement

The datasets presented in this study can be found in online repositories. The names of the repository/repositories and accession number(s) can be found below: NCBI - PRJNA895026.

## Ethics statement

The studies involving human participants were reviewed and approved by Clinical Research Ethics Committee of the Third Affiliated Hospital of Sun Yat-sen University. The patients/participants provided their written informed consent to participate in this study. The animal study was reviewed and approved by Institutional Animal Care and Use Committee of South China Agricultural University.

## Author contributions

LY designed the experiments and performed and interpreted the transcriptomic analysis. LY, LZ, and ZL wrote the first draft of the manuscript and incorporated revisions. LY and LZ collected liver samples for mRNA sequencing and validation. ZL performed the animal experiment. WX and QL collected and analyzed patient follow-up data. LP and CX guided the project and revised the manuscript. All authors contributed to the article and approved the submitted version.

## Footnotes

1
http://www.broadinstitute.org/gsea/index.jsp

2
http://www.omicshare.com

3
http://www.genemania.org

## Abbreviations

ACLF: acute-on-chronic liver failure
HBV-ACLF: hepatitis B virus-related ACLF
DEGs: differentially expressed genes
CHB: chronic hepatitis B
NCs: normal controls
HBV: hepatitis B virus
PBMCs: peripheral blood mononuclear cells
KEGG: Kyoto Encyclopedia of Genes and Genomes
GSEA: gene set enrichment analysis
FGF19: fibroblast growth factor 19
ADCY8: adenylate cyclase 8
KER17: keratin 17
mRNA-seq: mRNA sequencing
qRT-PCR: quantitative real-time-polymerase chain reaction
HBsAg: hepatitis B surface antigen
APASL: Asian pacific association for the study of the liver
IHC: immunohistochemical
H&E: hematoxylin-eosin
M&T: Masson’s trichrome
S&R: Sirius red
AST: aspartate aminotransferase
ALT: alanine aminotransferase
ALP: alkaline phosphatase
TBILI: total bilirubin
GGT: gamma-glutamyl transferase
PT: prothrombin time
INR: international normalized ratio
ALB: albumin
PCA: principal component analysis
CAMs: cell adhesion molecules
SFTA2: Surfactant Associated 2
GOLGA6B: Golgin A6 family member B
KLF1: KLF transcription factor 1
SCTR: secretin receptor
SFTA2: surfactant associated 2
ARHGAP40: Rho GTPase activating protein 40
SLC30A2: solute carrier family 30 member 2
PSORS1C1: psoriasis susceptibility 1 candidate 1
HCM: hypertrophic cardiomyopathy
HSC: hepatic stellate cell.
