# Supplementary material for: Human liver tissue transcriptomics revealed immunometabolic disturbances and related biomarkers in hepatitis B virus-related acute-on-chronic liver failure
Source: Front Microbiol. 2022 Dec 1;13:1080484. doi: 10.3389/fmicb.2022.1080484 (PMC9752073; doi:10.3389/fmicb.2022.1080484)
Supplement: Supplementary file 1 [file Data_Sheet_1.docx]

**SUPPLEMENTARY MATERIALS**

[**Supplementary Information**](https://static-content.springer.com/esm/art%3A10.1038%2Fsrep20759/MediaObjects/41598_2016_BFsrep20759_MOESM2_ESM.pdf)

**Supplementary Table 1:**

**Primers used in qRT‒PCR of human liver tissues and mouse ACLF model**

| **Target** | **Gene ID** | **5′Forward3′** | **5′Reverse3′** |
| --- | --- | --- | --- |
| HS-ACTB | 60 | CATGTACGTTGCTATCCAGGC | CTCCTTAATGTCACGCACGAT |
| HS-FGF19 | 9965 | ATGGCTACAATGTGTACCGATC | AGAAAGCCTCTGTTCTTGTACA |
| HS-ADCY8 | 114 | CATCGCTATGAGAACGTCAGTA | AGTCCCCCAGGATTTTAATACG |
| HS-KRT17 | 3872 | TGAGATCAATGTGGAGATGGAC | CTGTCTTGCTGAAGAACCAATC |
| MS-Actb | 11461 | CACTGTCGAGTCGCGTCCA | TGACCCATTCCCACCATCAC |
| MS-FGF15 | 14170 | GTCGCTCTGAAGACGATTGCCA | CAGTCTTCCTCCGAGTAGCGAA |
| MS-ADCY8 | 11514 | CAGTTCCACCGCATCTACATCCATC | GAGTTCGTTGAGCATCCTGACCAG |
| MS-KRT17 | 16667 | GCAGCAGAACCAGGAGTACAAGATC | GCATCCTCTCCCTCCAGCAGAC |

**Supplementary Table 2:**

**Antibodies**

| **Name** | **Source** | **Cat. No**. | **Species** |
| --- | --- | --- | --- |
| FGF19 | Abcam, Cambridge, UK | ab225942 | Rabbit |
| ADCY8 | Bioss, Massachusetts, USA | bs-3925R | Rabbit |
| KRT17 | SAB, Maryland, USA | 40822 | Rabbit |

**Sample preparation for mRNA-seq**

For mRNA-seq, liver tissues were collected from patients and donors and snap-frozen in liquid nitrogen, and total RNA was extracted using TRIzol reagent (15596018, Thermo Fisher Scientific, Inc., USA). Sequencing libraries were prepared according to the manufacturer’s instructions (TruSeq® RNA LT Sample Prep Kit v2, Illumina, San Diego, CA) with steps including purifying and fragmenting mRNA, synthesizing first-strand cDNA, synthesizing second-strand cDNA, performing end repair by adenylating the 3' ends, ligating adapters, and enriching DNA fragments. The pooled library consisted of sequences with lengths of approximately 250 nucleotides. The library was sequenced using the HiSeq 2500 sequencing system (Illumina).
